# The effect of Apremilast on signal transduction and IL-10 production in CD39high regulatory B cells in patients with psoriatic arthritis

**DOI:** 10.31138/mjr.29.1.59

**Published:** 2018-03-19

**Authors:** Lazaros I. Sakkas, Athanasios Mavropoulos, Efterpi Zafiriou, Aggeliki Roussaki-Schulze, Dimitrios P. Bogdanos

**Affiliations:** 1 Department of Rheumatology and Clinical Immunology and; 2 Dermatology, Faculty of Medicine, School of Health Sciences, University of Thessaly, Larissa, Greece

**Keywords:** Apremilast, CD39, psoriatic arthritis, psoriasis, regulatory B cells, T cells

## Abstract

**Background.:**

IL-10-producing regulatory B cells (Bregs) are of great importance in autoimmunity, as they inhibit proinflammatory T cells. We have shown that IL-10-producing Bregs in psoriatic arthritis(PsA) were decreased and inversely correlated with IFNγ+T cells (TH1 cells) and IL-17+ T cells (TH17 cells). B cells with overexpression of CD39 have also inhibitory effects on proinflammatory T cells.

**Preliminary results.:**

Our preliminary data showed that Apremilast, a phosphodiesterase-4(PDE-4) inhibitor, used in the treatment of PsA and psoriasis (Ps) increased IL-10-producing Bregs and reduced IFNγ+CD3+ T cells and IL-17+CD3+ T cells. We also found reduced activation of p38MAP kinase and the transcription factor STAT3, two important signaling pathways of IL-10 production, in PsA.

**Specific Aims.:**

The aim of this research proposal is to study for the first time the immunomodulatory effect of Apremilast on signaling pathways in peripheral blood mononuclear cells (PBMCs) and CD39high B cells in PsA and Ps.

**Methods.:**

We will study CD39 expression in B cells from patients with PsA and Ps before and after Apremilast treatment and their relation to IFNγ+ and IL-17+ T cells. Activation of CREB (cAMP response element-binding protein), STAT3, and p38MAPK in PBMCs and CD39high B cells from patients with PsA and Ps before and after Apremilast. The effect of CD39high B cells on T cell IFNγ and IL-17 production will also be studied.

**Significance.:**

This study will elucidate the molecular pathways of Apremilast and better define Bregs in PsA and Ps.

## INTRODUCTION

In recent years, a number of studies have shown that the deficiency of regulatory B cells (Bregs) is associated with the predominance of autoreactive B and T cells in autoimmune diseases (Bregs).^1^ Bregs, phenotypically divided into CD19(+)CD24(hi)CD38(hi) (transitional Bregs) and CD19(+)CD24(hi)CD27(+) (memory Bregs), inhibit proinflammatory cells mainly through interlukin-10 (IL-10).^2^ We have recently reported that IL-10+Bregs are deficient in systemic sclerosis (SSc), psoriatic arthritis (PsA), and psoriasis (Ps).^3,4^ PsA and Ps share many genetic and clinical features, so that one may consider them as one disease with different manifestations.^5^

The anti-inflammatory properties of IL-10 and its immunoregulatory action on proinflammatory T and B cells are well-documented.^6^ Bregs play a critical role in the maintenance of balance between regulatory T cells (Tregs) and proinflammatory Th1, Th17 cells.^7,8^ Bregs induced *in vitro* can subsequently induce naïve T cells into Tregs (CD25+, FoxP3+) producing IL-10.^9^

Recent studies have shown that overexpression of CD39 is also a marker of Bregs,^10^ and the suppression of T cells has been associated with increased expression of CD39 on cell membrane of B cells.^11^ Indeed, CD39high B cells co-cultured with autologous T cells inhibited T cell activation and expansion and increased IL-10 production.^10^ Tregs also overexpress CD39.^12^ CD39 is an ectonucleoside triphosphate diphosphohydrolase which is expressed on the surface of lymphocytes and catalyzes hydrolysis of ATP thus producing adenosine (ADO). ADO acting on its receptor activates adenylcyclase, which in turn catalyses ATP into cyclic AMP(cAMP) and leads to inhibition of T cell function.^13^ cAMP through the action of phosphodiestarse-4(PDE4) catalyzes the conversion of cAMP to AMP.

Apremilast inhibits PDE-4 and thus increases cAMP levels.^14^ Increased cAMP levels activate protein kinase A (PKA), up-regulate cAMP-response element(CRE)-containing genes, activating transcription factor-1 (ATF-1), while they inhibit nuclear factor-kappa B (NF-kB) involved in inflammatory responses.^14^ In lymphocytes, IL-10 production is regulated by a number of transcription factors, such as signal transducers and activators of transcription (STAT), cAMP-response element binding protein(CREB), NF-kB, activator proteins, including ATF-1.^15^

cAMP via PKA/CREB increases CD39 expression.^16^ It is worth noting that IL-35-stimulated CD39+ CD4+ T cells protected against collagen-induced arthritis via IL-10 production.^17^

## AIMS

Our research hypothesis is that Apremilast may directly increase IL-10 production and indirectly increase expression of CD39 in B cells and T cells, thus promoting their immunoregulatory capabilities. This may be mediated by transcription factors CREB, STAT3, p38MAK, and ERK1/2 MAPK.

## SPECIFIC AIMS

Based on the above discussion, we plan to study:

1. The expression of CD39 in B cells from patients with PsA and Ps and its relation to IFNγ+CD3+ T cells and IL-17+CD3+ T cells at baseline and after initiation of Apremilast treatment.
2. Activation (phosphorylation) of CREB, STAT3, p38MAPK in peripheral blood mononuclear cells (PBMCs) and purified CD39high B cells from patients with PsA and Ps at baseline and after Apremilast treatment.
3. Activation (phosphorylation of CREB, STAT3, p38MAPK in purified CD39high B cells from healthy controls, after stimulation with stimulants that induce IL-10 production with or without Apremilast *in vitro*.
4. The effect of CD39high B cells on the expression of IFNγ and IL-17 in co-cultures with T cells with or without Apremilast *in vitro*.

The expression of CD39 in B cells and the phosphorylation of signal transducers/transcription factors will be studied with flow cytometry and the use of appropriate monoclonal antibodies and protocols specifically designed for small cell populations as described by our group.^3^ Cell cultures will be carried out as described.^3^

## PATIENTS AND METHODS

### Patients

Patients with PsA (n=15), Ps(n=15) and healthy controls(n=15) will be included in this study. Patients with PsA and Ps, followed up at the outpatient clinics of the Departments of Rheumatology and Clinical Immunology and Dermatology, respectively, will participate in the study after written informed consent, and approval by the Scientific Committee of the University General Hospital of Larissa.

Peripheral blood samples (20 mL) will be collected from patients at baseline and at month 3 and 6 after Apremi-last initiation. Peripheral blood mononuclear cells (PBMCs) and serum are isolated and kept in liquid nitrogen tanks until used. We already have in biobanking facility PBMCs for 20 patients ready to use.

### Methods

Lymphocytes will be cultured in RPMI 1640 with serum (5–10%) at 37° C. B cells be characterized by antibodies to surface markers CD19, CD24, CD38, and CD27, and naïve and memory B cells, and transitional and memory Bregs with be measured. CD39high Bregs will be characterized by staining with anti-CD39 monoclonal antibody and intracellular staining for IL-10. From another cryovial PBMCs, naïve and memory B cells will be separated with magnetic beads (Miltenyi-B cell negative selection). Similarly, CD4+ T cells will be separated for PBMCs.

CD39high B cells or PBMCs will be cultured with or without polyclonal IgM (BCR activation) bacterial CpG (ODN2006 –TLR-9 activation), bacterial LPS (TLR-4 activation, PMA, ionomycin, in order to activate/differentiate B cells into IL-10 production in the presence or no Apremilast. Incubation times will vary from 24 hours to 5 days. CD39high B cells will be cultured with autologous CD4+Tcells with or without Apremilast, and the effect on T cells will be assessed with T cell proliferation assays or with measurement of T cell IFN-γ production. For intra-cellular cytokine production, brefeldin A, an inhibitor of protein transport, will be added, and cells will be stained for membrane markers. Then after treatment with saponin solution, cells will be stained for intracellular cytokines with appropriate monoclonal antibodies.

Phosphorylation of CREB, ERK1 / 2, ρ38 MAPK and STAT-3 will be carried out with BD^TM^ Phosflow protocol, as described by our group (Mavropoulos). In brief, cells will be left to rest in 1% fetal calf serum (FCS) for 2 hours and then they will be stimulated with CpG-DNA (ODN2006) and / or anti-goat IgM polyclonal antibody (BCR stimulant) and anti-CD40 antibody. To measure baseline kinase phosphorylation, in a parallel culture cells will be left without stimulation. For cell fixation, cells will be treated with paraformaldehyde (2%) at 37° C for 15 min. Then, after washing, cells will be permeabilized with 1 ml 75% (ν / ν) methanol (Fisher Scientific, Pittsburgh, PA) in TBS. Then cells will be hydrated for 30–60 min. After washing, cells will be treated with FcR block at room temperature for 15 min to block Fc receptors. After washing, cells will be resuspended in 2% BSA / TBS (w/v) and will be incubated with conjugated fluorochromes. The following monoclonal antibodies will be used for phosphor-flow: CREB (pS133), phospho-p38 MAPK (T180 / Y182) καı Stat3 (pS727) (BD Biosciences). According to the manufacturer, Stat-3 is phosphorylated at serine 727 (S727) by MAPK.

## SIGNIFICANCE

This study is novel, and the hypothesis is based on preliminary data. Much of biological material is available for the initiation of the study. The identification of molecular pathways though which Apremilast exert its actions in PsA and Ps may reveal new therapeutic strategies for these diseases. Finally, our study will help better define the role of various Bregs, a key player in autoimmune rheumatic diseases.

## References

[1] Mauri C Menon M. Human regulatory B cells in health and disease: therapeutic potential. J Clin Invest. 2017 1;127:772–9. 28248202 10.1172/JCI85113 PMC5330739

[2] Kalampokis I Yoshizaki A Tedder TF. IL-10-producing regulatory B cells (B10 cells) in autoimmune disease. Arthritis Res Ther. 2013;15 Suppl 1:S1. doi: 10.1186/ar3907. 10.1186/ar3907 PMC3624502 23566714

[3] Mavropoulos A Simopoulou T Varna A Liaskos C Katsiari CG Bogdanos DP Sakkas LI. Breg Cells Are Numerically Decreased and Functionally Impaired in Patients With Systemic Sclerosis. Arthritis Rheumatol 2016;68:494–504. 26414243 10.1002/art.39437

[4] Sakkas LI Bogdanos DP. Are psoriasis and psoriatic arthritis the same disease? The IL-23/IL-17 axis data. Autoimmun Rev. 2017;16:10–5. doi: 10.1016/j.autrev.2016.09.015. 27666819 10.1016/j.autrev.2016.09.015

[5] Mavropoulos A Varna A Zafiriou E Liaskos C Alexiou I Roussaki-Schulze A IL-10 producing Bregs are impaired in psoriatic arthritis and psoriasis and inversely correlate with IL-17- and IFNγ-producing T cells. Clin Immunol. 2017;184:33–41. 28461105 10.1016/j.clim.2017.04.010

[6] Lampropoulou V Calderon-Gomez E Roch T Neves P Shen P Stervbo U Suppressive functions of activated B cells in autoimmune diseases reveal the dual roles of Toll-like receptors in immunity. Immunol Rev 2010;233:146–61. 20192998 10.1111/j.0105-2896.2009.00855.x

[7] Carter NA Rosser EC Mauri C. Interleukin-10 produced by B cells is crucial for the suppression of Th17/Th1 responses, induction of T regulatory type 1 cells and reduction of collagen-induced arthritis. Arthritis Res Ther. 2012 8;14:R32. 10.1186/ar3736 PMC3392827 22315945

[8] Flores-Borja F Bosma A Ng D Reddy V Ehrenstein MR Isenberg DA Mauri C. CD19+CD24hiCD38hi B cells maintain regulatory T cells while limiting TH1 and TH17 differentiation. Sci Transl Med 2013;5:173ra23. 10.1126/scitranslmed.3005407 23427243

[9] Kessel A Haj T Peri R Snir A Melamed D Sabo E Toubi E. Human CD19(+)CD25(high) B regulatory cells suppress proliferation of CD4(+) T cells and enhance Foxp3 and CTLA-4 expression in T-regulatory cells. Autoimmun Rev. 2012;11:670–7. 22155204 10.1016/j.autrev.2011.11.018

[10] Figueiro F Muller L Funk S Jackson EK Battastini AM Whiteside TL. Phenotypic and functional characteristics of CD39high human regulatory B cells (Breg). Oncoimmunology 2016 2;5(2):e1082703. 27057473 10.1080/2162402X.2015.1082703 PMC4801473

[11] Saze Z Schuler PJ Hong CS Cheng D Jackson EK Whiteside TL. Adenosine production by human B cells and B cell-mediated suppression of activated T cells. Blood. 2013;122:9–18. 23678003 10.1182/blood-2013-02-482406 PMC3701906

[12] Schuler PJ Saze Z Hong CS Muller L Gillespie DG Cheng D Human CD4+ CD39+ regulatory T cells produce adenosine upon co-expression of surface CD73 or contact with CD73+ exosomes or CD73+ cells. Clin Exp Immunol 2014;177:531–43. 24749746 10.1111/cei.12354 PMC4226604

[13] Bopp T Becker C Klein M Klein-Hessling S Palmetshofer A Serfling E Cyclic adenosine monophosphate is a key component of regulatory T cell-mediated suppression. J Exp Med 2007;204:1303–10. 17502663 10.1084/jem.20062129 PMC2118605

[14] Sakkas LI Mavropoulos A Bogdanos DP. Phosphodiesterase 4 Inhibitors in Immune-mediated Diseases: Mode of Action, Clinical Applications, Current and Future Perspectives. Curr Med Chem. 2017;24:3054–67. 28554321 10.2174/0929867324666170530093902

[15] Iyer SS Cheng G. Role of interleukin 10 transcriptional regulation in inflammation and autoimmune disease. Crit Rev Immunol 2012;32:23–63. 22428854 10.1615/critrevimmunol.v32.i1.30 PMC3410706

[16] Liao H Hyman MC Baek AE Fukase K Pnsky DJ. cAMP/CREB-mediated transcriptional regulation of ectonucleoside triphosphate diphosphohydrolase 1 (CD39) expression. J Biol Chem 2010;285:14791–805. doi: 10.1074/jbc.M110.116905. 20178980 10.1074/jbc.M110.116905 PMC2863166

[17] Kochetkova I Golden S Holderness K Calls G Pascula DW. IL-35 stimulation of CD39+ regulatory T cells confers protection against collagen II-induced arthritis via the production of IL-10. J Immunol 2010;184:7144–53. doi: 10.4049/jimmunol.0902739. 20483737 10.4049/jimmunol.0902739 PMC3145775

